# A Patient’s Six-Month Journey From Low Sodium to Blue Toes to Stroke: Non-infective Thrombotic Endocarditis Due to Non-small Cell Lung Cancer

**DOI:** 10.7759/cureus.23235

**Published:** 2022-03-16

**Authors:** Jocelyn McCullough, Joseph McCullough, Alan Kaell

**Affiliations:** 1 Medicine, Zucker School of Medicine at Hofstra, Hempstead, USA; 2 Hospital Medicine, Zucker School of Medicine at Hofstra, Hempstead, USA

**Keywords:** cancer anticoagulation, cerebrovascular stroke, syndrome of inappropriate antidiuretic hormone secretion, non small cell lung cancer, non-bacterial thrombotic endocarditis

## Abstract

We report a patient's journey with a four-year history of hypertension (HTN) and hyperlipidemia (HLD), stable on beta-blocker and statin, monitored every six months by alternating visits between her cardiologist and primary care physician (PCP) in North Carolina (NC). Six months before relocating to New York (NY) she had been informed about incidental severe hyponatremia during her last outpatient visit, the need for repletion with sodium chloride tablets, and the critical importance of prompt follow-up to rule out malignancy by starting with a chest X-ray. She opted not to follow instructions, continued cigarettes, and decided to spend the summer season with her son in NY. Six months after being told of her low sodium, she presented to our NY hospital with an acute, painful right foot blue toe syndrome. During the ischemic right foot evaluation, she was discovered to have adenocarcinoma of the right lung (stage 4) and a normal transthoracic echocardiogram (TTE). Heparin was initiated and thromboembolectomy with an endovascular bovine patch to revascularize the foot was successful, and post-procedure apixaban was started.

Hyponatremia was attributed to the syndrome of inappropriate antidiuretic hormone release (SIADH) secondary to non-small cell lung cancer (NSCLC). The serum sodium was stabilized, and the patient was discharged with a plan for outpatient follow-up with the cardiologist and oncologist within two weeks for hypertension, hyperlipidemia, hyponatremia, and management of stage 4 NSCLC. During her cardiology follow-up, 10 days after discharge, complaints of mild dyspnea on exertion (DOE) prompted an ECG (electrocardiogram) that revealed new T wave inversions in leads V3-6, and the patient was readmitted for non-ST elevation myocardial infarction (NSTEMI) evaluation. On day one of the readmission troponins were negative with normal ejection fraction (EF) on TTE and an acute 2 g/dl hemoglobin (Hb) drop with melena. This led to discontinuation of anticoagulation, initiation of intravenous (IV) pantoprazole, and endoscopy (EGD) which revealed gastritis. On the third day, she developed sudden expressive aphasia. Computed tomography (CT) of the head did not show any bleed but same-day magnetic resonance imaging (MRI) demonstrated multiple evolving acute infarcts. Transesophageal echocardiogram (TEE) demonstrated two large, mobile masses on the mitral valve consistent with vegetative endocarditis. Cultures for bacteria, fungi, and evaluation for organisms associated with culture-negative acute bacterial endocarditis/subacute bacterial endocarditis were unrevealing, thus confirming malignancy-associated non-infectious thrombotic endocarditis or non-bacterial thrombotic endocarditis (NBTE).

Gastrointestinal (GI) bleeding ceased, and the patient initially started on a heparin drip and transitioned to enoxaparin as lifelong anticoagulation for malignancy-associated NBTE. She recovered neurologically and was given pembrolizumab. At her recent 15-month visit she continued to have no residual neurological impairments, however, new positron emission tomography (PET) detected metastasis to the liver, lung, and adrenals which prompted evaluation for hospice care. We, therefore, emphasize the need for timely diagnosis of NBTE and prompt initiation of anticoagulation in suitable patients to prevent complications such as in our patient. Additionally, hyponatremia secondary to SIADH in NSCLC is a poor prognostic indicator of overall survival.

## Introduction

Hyponatremia due to syndrome of inappropriate antidiuretic hormone release (SIADH) as a manifestation of non-small cell lung cancer (NSCLC)

Hyponatremia attributed to paraneoplastic SIADH is more commonly seen in patients with small-cell lung cancer (SCLC) compared to all other cancers [1]. The incidence of SIADH over a nine-year period, although higher in patients with SCLC at 26%, is elevated at 16% in those with NSCLC [2]. Paraneoplastic SIADH whether detected in either NSCLC or SCLC usually occurs in stage 4 and portends a poor prognostic indicator of overall survival. In general, there is a low likelihood that prompt recognition of the SIADH due to NSCLC six months earlier would have revealed a more favorable stage. 

Non-infective thromboembolic endocarditis

The original term marantic endocarditis referred to sterile thromboembolic endocarditis seen in patients with malignancy. The term non-bacterial thrombotic endocarditis (NBTE) has replaced “marantic”, however, culture-negative endocarditis has been attributed to both difficult organisms to culture and sterile thrombotic valvular vegetations seen in systemic lupus erythematosus (Libman Sacks endocarditis), antiphospholipid syndromes (APS), and malignancy with or without APS [3-8]. Causes of infective endocarditis that are “culture” negative include: prior administration of antibiotics, organisms that require a longer period of time to grow in the culture media, e.g., *Cutibacterium* spp. and the HACEK bacteria group (*Haemophilus, Aggregatibacter, Cardiobacterium, Eikenella, and Kingella*), and fastidious pathogens like *Aspergillus* species, *Brucella* species, *Coxiella burnetii*, or *Chlamydia* species. Some infective endocarditis is defined not by culture but by serology or polymerase chain reaction (PCR), such as *Tropheryma whipplei*. Such patients may be erroneously classified as "culture-negative" endocarditis [9-12]. 

We prefer the less confusing term “non-infective endocarditis” [13] when referring to truly sterile valvular vegetations but will use the traditional literature term and acronym NBTE with the awareness that “culture-negative” may not necessarily define non-infective endocarditis. Therefore, NBTE will be used in the sterile sense of non-infective endocarditis. Such sterile vegetations may occur if damage to the endothelial lining of the heart valves from trauma, circulating immune complexes or hypercoagulable states occurs. A cascade of events can be initiated that trigger the deposition of sterile thrombotic vegetations along with these valves. Most vegetation occurs on the mitral or aortic valves since they are subjected to higher pressure gradient turbulent blood flow compared to the right-sided heart valves. The mucinous substances, especially from pancreatic mucinous adenocarcinoma, may also lead to valvular vegetations [14]. These vegetations are friable and tend to cause more thromboembolic phenomena compared to those of infective endocarditis [15]. 

## Case presentation

A 66-year-old female, 30 pack-year cigarette smoker, presented to our New York hospital with the acute one-day onset of painful right blue toes. Six months prior to her presentation, while still residing in North Carolina (NC), she was informed of severe hyponatremia during her routine lab for her primary care physician (PCP) six-month follow-up for hypertension on beta-blocker and hyperlipidemia on statins. She did not have any symptoms of hyponatremia such as confusion or gait disturbance and was not on diuretics or have a history of hypothyroidism. The patient was urged to follow up with a chest X-ray and CT chest to rule out lung malignancy and began taking 1-gram sodium chloride tabs three times a day as she preferred to avoid inpatient treatment. Unfortunately, the patient did not follow up with imaging studies and moved out of state and did not establish care with a new provider. 

The patient presented to our hospital with right toe discoloration. On physical examination, the patient’s right foot was warm but had bluish discoloration of the right great toe and fifth toe with mottling of the right foot. Pulses were worse with elevation and prolonged capillary refill time >2 seconds. On cardiac examination, the patient had a regular heart rate without any murmur. The patient had an ankle brachial index which showed 0.89 on the right foot and 1.09 on the left. No segmental pressure gradient was detected. Toe brachial index measures 0.97 on the left and it was unobtainable on the right due to cyanotic digits. Pulse volume recording (PVR) waveforms were mildly diminished in amplitude throughout the right leg compared to the left. Right digital waveforms were non-pulsatile. Mild arterial inflow limitation was seen in the right lower extremity compatible with known common femoral and superficial femoral artery stenosis, there was also mild arterial inflow limitation and some small vessel disease.

**Figure 1:**
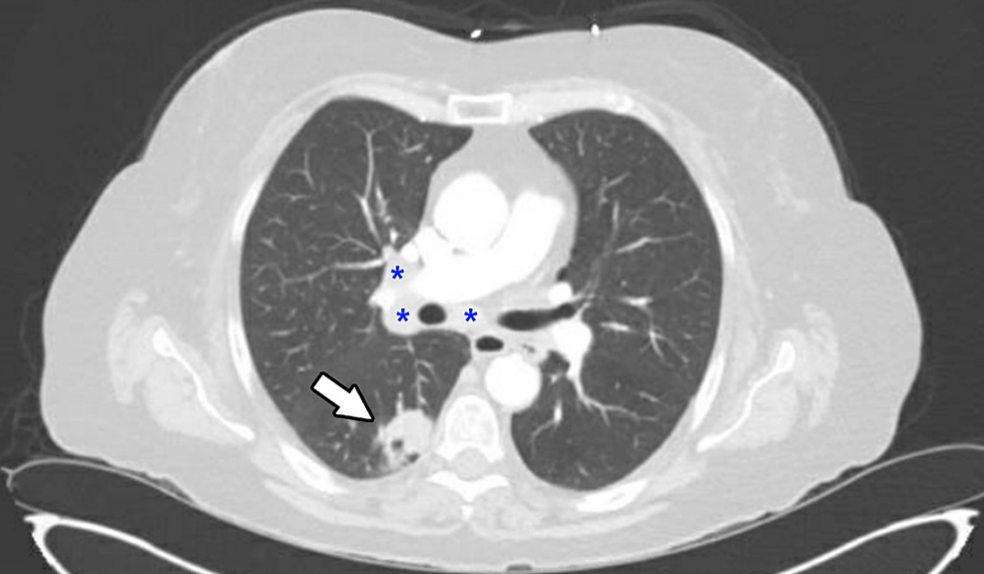
Computed Tomography of the Chest with Intravenous Contrast White arrow: Adenocarcinoma of the superior segment of the right lower lobe Blue asterisks: Right hilar adenopathy and subcarinal lymph node

The patient also had a CT scan of the lower extremities with intravenous (IV) contrast (iohexol) which showed high-grade stenosis of the proximal right superficial femoral artery while below knee runoff was limited to contrast. The CT chest, abdomen, and pelvis with IV contrast were added since chest X-ray on presentation showed concerns for a right hilar lung mass, The CT chest showed a 2.8 cm enlarging mass in superior segment of the right lower lobe and right hilar adenopathy and enlarged lymph nodes in the right pretracheal space (Figure 1). The patient was seen by vascular surgery and had an embolectomy of the right superficial femoral artery, an endarterectomy of the common femoral artery with bovine patch repair, and started on aspirin and apixaban. Core needle biopsy of the lung mass revealed poorly differentiated adenocarcinoma. The patient’s urine sodium was >40 meq and the urine osmolality was 281 mOsm confirming SIADH. The patient was discharged home and asked to follow up with a cardiologist for management of hypertension (HTN)/hyperlipidemia (HLD) and an oncologist for the newly diagnosed lung adenocarcinoma.

During her outpatient two-week follow-up visit with the cardiologist, the patient was complaining of shortness of breath (SOB) and an ECG was obtained which showed new T wave inversions in leads V3-V6 and she was sent to the emergency room for possible non-ST elevation myocardial infarction (NSTEMI). 

On admission day one, the patient’s troponin was 0.09 ng/ml ( nl <0.04 ng/mL) normal EF of 55% on transthoracic echocardiogram (TTE). NSTEMI was ruled out and the patient’s SOB was attributed to severe anemia secondary to an acute upper gastrointestinal (GI) bleed with melanotic guaiac positive stool and a drop in hemoglobin (Hb) from 9.2 gm/dl to admission hemoglobin of 7.4 gm/dl. She received one unit of packed red blood cells (RBC) and was started on an intravenous pantoprazole drip for concerning GI bleed. An endoscopy revealed gastritis without any evidence of ulcers, *Helicobacter* ​*Pylori,* or additional malignancy. Apixaban was held on day one. 

On the morning of day three, she developed sudden expressive aphasia, 72 hours after her last apixaban dose at home. A head CT performed that day did not show acute bleed, and an MRI obtained within hours showed multiple evolving acute infarcts including the left parietal lobe, bilateral occipital lobes, bilateral cerebellar hemispheres, frontoparietal hemispheres including centrum semiovale which suggested multiple embolic events. Since two previous TTEs, one from the 1st admission for ischemic toes and one performed during this 2nd admission for SOB did not reveal any valvular vegetations, the embolic stroke prompted a transesophageal echocardiogram (TEE) procedure. This confirmed two large highly mobile masses on the mitral valve, the largest one measuring 1.4 cm by 0.6 cm attached to A1 and P1 scallops of the mitral valve leaflets on the atrial aspect (Video 1). Blood cultures were subsequently obtained and were found to be negative after 48 hours, and a diagnosis of non-infective thrombotic endocarditis (NBTE) attributed to her NSCLC was confidently made. 

**Video 1:** Vegetations of A1 and P1 scallops of the mitral valve leaflets

Prior to discharge, the patient’s Hb was stable at 9.1 gm/dl and stools were normal without occult blood. For her NBTE, she was started on heparin drip on day three without further GI bleeding and was later switched to a therapeutic dose of enoxaparin prior to discharge. She had complete resolution of her aphasia on day five after initiation of heparin drip. The patient followed up with her oncologist and a PD-1 (programmed death-1) expression was found to be >90% and hence started on pembrolizumab. Fortunately, she did not have any other thromboembolic events or GI bleeding for the next 15 months with enoxaparin. However, a recent PET scan showed innumerable new bilateral pulmonary nodules, left adrenal foci and right hepatic lobe foci, and left ischial tuberosity foci demonstrating progressive metastatic disease and the patient elected home hospice at that time. 

## Discussion

Hyponatremia due to SIADH as a manifestation of NSCLC

Hyponatremia linked to different kinds of cancers is most commonly linked with lung cancer, especially SCLC but also NSCLC as in our patient. When detected prior to malignancy recognition and chemotherapy treatment, low sodium is typically attributed to the increase in ectopic release of arginine vasopressin or secondary SIADH. However, after diagnosis and treatment commences hyponatremia may also be related to side effects from certain chemotherapy and depletion of electrolytes from vomiting and diarrhea. 

However, hyponatremia has been well established as an independent prognostic indicator of overall survival in patients diagnosed with adenocarcinoma with lower sodium associated with poorer outcomes [2]. In a Danish population-based cohort study from 2021 on hyponatremia in lung cancer, data extracted from the Danish Lung Cancer Registry and combined with pre-treatment sodium level from the clinical laboratory information system demonstrated that among patients with NSCLC 53% had adenocarcinoma and 85% were current/ former smokers. A sodium level below 135 mmol/l was found in 16 % of all patients with NSCLC. Mild hyponatremia was found in 69 % of the patients, moderate hyponatremia in 27 % and severe hyponatremia (< 125 mmol/l) in 4% of the patients. A higher frequency of patients with hyponatremia had an advanced stage of the disease. Hyponatremia remained an independent predictor of overall survival. With increasing hyponatremia severity there was a decline in overall survival. 

On review of the literature there does not seem to be a correlation or direct association of risk between malignant SIADH hyponatremia and the presence of non-infective thromboembolic endocarditis. We can only speculate that if our patient complied with the chest X-ray and high-resolution computer tomography (HRCT) of her chest six months prior to her presentation of thromboembolic ischemic right foot, that perhaps a more favorable stage NSCLC would have been discovered. 

Non-infective thromboembolic endocarditis

Autopsy studies in patients with malignancy often demonstrate valvular vegetations consistent with non-infective endocarditis [3]. A 2019 Italian study was an autopsy study of both infective and non-infective endocarditis of 814 patients that looked at the clinical manifestations and basically found them indistinguishable [15]. Certainly, such thromboembolic clinical events in patients with a known malignancy (particularly adenocarcinoma of the lung or pancreas) should prompt TTE evaluation. If clinical suspicion is high and initial TTE is negative, physicians should still proceed with a TEE to help identify vegetations that may be missed on TTE, which is known to be less sensitive imaging tool. While murmur is also not a common occurrence, a new soft systolic murmur in a patient with cancer should also prompt evaluation for NBTE. Diagnosis requires imaging and the preferred test being TEE. A biopsy is not done routinely, but samples can be studied on patients who require valvular replacement and typically do not show malignant cells or infection, but fibrin thrombus [3]. 

## Conclusions

Patients with a history of smoking and presenting with hyponatremia should have additional workup to rule out malignancy in addition to smoking cessation counseling. Unfortunately, our patient opted to ignore recommendations, perhaps due to denial. Any clinical presentation of thromboembolic events such as blue toes should prompt clinicians to investigate further that includes timely TTE/TEE in those patients who have significant risk factors like in our patient. Patients with thromboembolic events secondary to malignancy-related endocarditis should be managed with enoxaparin to prevent further thromboembolic phenomena. Our patient with NBTE attributed to stage 4 NSCLC was started on enoxaparin and treated with pembrolizumab experienced 15 months survival free of any further thromboembolic phenomena. A timelier diagnosis of her NSCLC and institution of therapy may have precluded the emergence of NBTE with clinical thromboembolic phenomena to her right foot and brain. 
